# Improvement of Lithium-Metal Batteries by Addition of a Low Concentration of Organic Molecules

**DOI:** 10.1021/acsami.5c01585

**Published:** 2025-05-01

**Authors:** Roy Marrache, Emanuel Peled

**Affiliations:** School of Chemistry, Faculty of Exact Sciences, Tel Aviv University, Tel Aviv 69978, Israel

**Keywords:** lithium, batteries, SEI, liquid electrolyte, additives

## Abstract

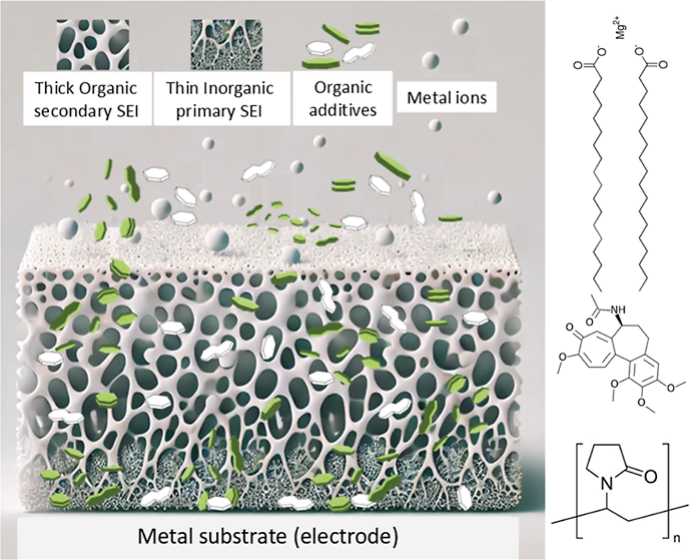

Batteries are already considered as one of the main issues if addressing global warming, and humans generate green energy while electrifying all their services. Increasing the ability to store energy for long periods of time will enable us to store solar and wind energy and use it where the sun or wind is not present. A known method for enhancing batteries is the use of additives to liquid electrolytes, which usually address the solid electrolyte interphase (SEI) properties, including Li conductivity, roughness of deposits, and chemical composition. Their reduction starts upon first contact between the metal and the electrolyte and at the first charge of a lithium-metal cell. A good property SEI is essential for a safe and long-lasting cell. Herein, we explore widely used organic molecules as electrolyte additives for different Li-based batteries and their effect on the properties of the SEI. The investigated systems were Li-metal||Cu and NiCoAl||Cu which exhibited a 11.3% and 1.4% improvement in average Coulombic efficiency, respectively, reaching above 99.0%.

## Introduction

Humans have caused dramatic atmospheric changes since the industrial revolution, involving increasing gas emissions, mainly caused by burning fossil fuels. Many options have been proposed to mitigate this issue and to try and create a reality where 21st century life could be achieved without any pollution causing atmospheric changes, not to mention negative emissions. Green electricity is already a readily available solution, using blade movement caused by the wind or photovoltaic solar panels. To complete this solution, accumulation of this energy is needed. Several solutions have emerged and been proposed over the years, including green H_2_, metal-based energy storage systems (such as Li and Na), supercapacitors, and more.^1−5^

Lithium-ion batteries (LIB) are currently used as the main source of energy for electric vehicles (EVs) and energy storage and portable devices.^1^ The whole world is moving toward increasing the use of EVs, through lithium batteries, constantly charged by renewable energies, mainly the sun and wind.^2^ LIBs have had a profound impact on modern society.^3^ Over the past 25 years, the specific energy of LIBs has steadily increased at the same time that their cost has decreased dramatically. However, these batteries are approaching their specific energy limits,^4^ while the energy storage market of EVs requires an even higher specific energy, to more than 500 watt kg^–1^ at the cellular level, and at a lower cost, below 100 USD (kW h)^−1^ at the cell level.^5^ The LIB is becoming more difficult to meet people’s needs in our electronics consumption era. Metallic lithium is considered a potential future anode material for rechargeable batteries with high energy density.^5^ Because lithium is a very light metal (0.534 gr cm^–3^), obtaining a very high specific capacity (3862 mA h g^–1^) (ten times that of graphite), and as it has the most negative standard electrochemical potential (−3.04 V vs SHE), lithium-metal batteries (LMBs) have higher energy density compared to LIBs and, thus, are an attractive next-generation energy storage system (ESS). In LMB systems, lithium deposits and dissolves during the charge and discharge processes, respectively. One of the major disadvantages of these systems is the excessive use of lithium which dramatically lowers the practical energy density of the cell. The main solution to address this issue, which is both less safe and more expensive, is the use of anode-free lithium-metal batteries (AFLMBs). In this configuration, the exact amount of lithium ions originated at the cathode will participate in the deposition/dissolution of lithium that occurs on the copper current collector. The absence of anodic volume and weight significantly increases the volumetric energy density and specific energy of the battery.

Many undesirable processes take place during the cycling of LMBs and AFLMBs. Many of these processes are related to the solid electrolyte interphase (SEI). The SEI starts to form instantly with an alkali metal/electrolyte contact and continues to form mainly during the first charge of the battery. The properties of the SEI are crucial for a safe long-lasting battery.^6^ The undesirable processes include the reduction of electrolyte components, capacity consumed by the repair of the SEI, formation of “dead Li”, and dendrite formation.^6^ One of the major reasons for the progression of such processes is uneven deposition of lithium on the anodic current collector that might result from a rough surface and concentration gradients.^7^ Many approaches have been suggested to overcome these issues. One of the most straightforward approaches is to add additives to liquid electrolytes and there is vast research in this area.^8,9^ Lee et al. developed an efficient electrolyte for a LMB (1 wt % adiponitrile in 0.8 m LiTFSI + 0.2 M LiDFOB + 0.05 M LiPF_6_ dissolved in a EMC/FEC 3:1 electrolyte, Li-metal||NiCoMnAl) using a bifunctional additive, adiponitrile, which demonstrated great cycling stability and a high Coulombic efficiency (CE) of >99.5% over 830 cycles even at a fast charge–discharge time of 2C with 1.8 mA h cm^–2^.^10^

The use of polyether-modified siloxanes^11^ or phenylenedimaleimide compounds^12,13^ has improved the cycling performance of various anodes including carbon and silicon. It was shown that these electrode additives were reduced prior to the solvent reduction to form a stable SEI on the electrode’s surface, as well as improving cycling performance compared to the cell without the additives.

Other approaches were suggested to improve alkali-metal batteries, both with liquid and solid electrolytes.^14−16^ These include the use of covalent organic frameworks to stabilize the metal,^17^ the use of nanocomposite current collectors for an anode-free solid-state lithium battery,^18^ or the use of nanostructures in liquid electrolytes which might be referred to as suspended electrolytes.^19−22^

Recently, our laboratory introduced a new family of additives in the form of metal-oxides nanoparticles (MONPs), which led to cyclability improvements.^20,21^ The addition of 0.5% In_2_O_3_ and ZnO NPs resulted in a 99.9% current efficiency while it took 46 cycles for a cell with the addition of 1% In_2_O_3_ NPs to lose 30% of its initial capacity, in the AFLMB (Cu||NiCoAl).^21^ The same cell without MONPs presented 97.1% CE and lost 30% of its initial capacity after only 12 cycles. Deeper investigation on AFLMBs with ZnO addition could be found elsewhere.^22^

In the graphical abstract, the chemical structure of the investigated additives is presented. These include colchicine (pCOL),^23^ polyvinylpyrrolidone (PVP),^24^ and Mg stearate (Mg-st).^25^ pCOL contains resonance rings and high content of oxygen, PVP is a polymer with amide groups, and Mg-st is a saturated amphiphilic molecule with Mg in the hydrophilic head.

Using a low concentration of organic additives to liquid electrolytes could open a new research approach for anode-free alkali-metal ESSs (but not only for them), where the anode is not fabricated pre-cell assembly (as it is done traditionally) but during the first charge of the battery. This research could open the door for investigation of new molecules as liquid electrolyte additives for different ESSs.

This work examines the effect of three different widely used organic molecules as additives for two different Li-metal systems: LMB and AFLMB. Our goal was to better understand the generated SEI and the effect on the properties of the cell. It was found that the addition of a mixture of the additives to the LMB greatly reduces charge transfer resistance (*R*_CT_) and increases the Coulombic efficiency (CE) by more than 10%. For the AFLMB configuration, the same organic mixture (OM) addition reduces the resistance of the bulk (*R*_b_) and increases the lifespan of the battery by more than 100%. Furthermore, it was found that magnesium stearate (Mg-st) has even a more pronounced improvement on the AFLMB configuration.

## Experimental Section

### Cell Materials, Dimensions, and Electrolyte

Coin cells (CR2032) were assembled in an Ar-atmosphere glovebox (0.1 ppm of H_2_O and O_2_, each) (MBraun).

Two sets of experiments were made and are composed of the following:

(a) two layers of Celgard 2400 separator (Celgard, USA) placed between a 12 mm-diameter NiCoAl (NCA, 8:1.5:0.5, ∼3.1 mA h cm^–2^) cathode (Tadiran Batteries Ltd.) and press-rolled 30 μm thick 15 mm-diameter copper foil (Schlenk). The electrolyte (Soul Brain) was composed of 0.95 M LiPF_6_ + 0.05 M Li bis(oxalato)borate (LiBOB) dissolved in EMC:DMC:FEC:PC 3:3:3:1 (v/v).
(b) Two layers of Celgard 2400 separator (Celgard, USA) placed between a 30 μm thick and 19 mm-diameter copper foil (Schlenk) and press-rolled 15 mm-diameter lithium foil (Rockwood Lithium). The electrolyte studied is 1 M lithium hexafluorophosphate (LiPF_6_, Solvionic) dissolved in ethylene carbonate:diethyl carbonate 1:1 (v/v) (EC:DEC).

### Organic Additives

The investigated materials were colchicine, PVP, and Mg-st. Pure colchicine (pCOL, 95%, Alfa Aesar) was vacuum-dried at 90 °C, PVP (M.W. 8000, Alfa Aesar) was vacuum-dried at 120 °C, and Mg-st (Mg-st, Riedel-de Haen) was vacuum-dried at 60 °C, all for 8 h. The organic mixture (OM) electrolytes were prepared by adding equal weights of the three additives relative to the weight of the electrolyte (for 1% OM electrolyte, 0.33% of each additive was added). The mixed electrolytes were magnetically stirred for 4 h before cell assembly. The concentrations were chosen similarly to other works.^10,20,21^

### Cell Assembly

Four stainless steel (SS) spacers and one SS spring were inserted in each coin cell, forcing contraction of the spring to apply a pressure of ∼5.3 atm on the cell (estimated from the calibration curve of the force-vs-contraction distance for the SS spring and the thickness of each component of the coin cell). The electrolyte volume in each cell was 60 μL. All the electrolytes were magnetically stirred for four hours before cell assembly. The cells rested under an OCV condition at 30 °C for 24 h before testing.

### SEI Characterization

ESEM micrographs were carried out with a Quanta 200 FEG ESEM. X-ray photoelectron spectroscopy (XPS) measurements were performed under UHV conditions (2.5 × 10^–10^ Torr base pressure) with the use of a scanning 5600 multitechnique system (PHI, USA). The samples were transported under an Ar atmosphere and were introduced into the UHV environment without exposure to air. The samples were irradiated with an Al Kα monochromatic source (1486.6 eV), and the emitted electrons were analyzed by a spherical capacitor analyzer with a slit aperture of 0.8 mm. Depth profiling was performed with a 4 kV Ar + ion gun (sputter rate was 56.3 Å min^–1^ on a SiO/Si reference). Copper electrodes from disassembled cells were washed twice in dimethylcarbonate (DMC) and were vacuum-dried at 50 °C for 12 h before analysis.

Cycling tests and electrochemical-impedance-spectroscopy (EIS) measurements were carried out with the use of a BioLogic BCS or VMP3. EIS measurements were made over a frequency range of 0.1 Hz to 1 MHz, and their spectra were analyzed by EC-Lab software.

## Results and Discussion

Anode-free NCA||Cu and Li||Cu cells were built with the following electrolytes: 0.95 M LiPF_6_ + 0.05 M LiBOB dissolved in EMC:DMC:FEC:PC 3:3:3:1 (v/v) and 1.0 M LiPF_6_ dissolved in EC:DEC 1:1 (v/v), with and without addition of 1% and 2% (w/w) organic mixture (OM), respectively.

NCA/Cu cells were cycled at C/10 (366 μA) where the charge cutoff voltage was 4.25 V and the discharge cutoff voltage was 3.0 V. Cycling stopped when the cell lost 30% of its initial capacity (cycle of 70% CR). Since the highest discharge capacity in the initial cycles was obtained at a different cycle for each cell type (range from cycle 2 to 4), the initial discharge capacity for each cell was calculated as the average discharge capacity of cycles 2, 3, and 4. The Coulombic efficiency (CE, defined as ) was calculated as an average from cycle 2 until the cycle at which the cell reached 70% CR (for convenience, we define the cycle number at 70% CR as “cycle 70% CR”).

**Figure 1:**
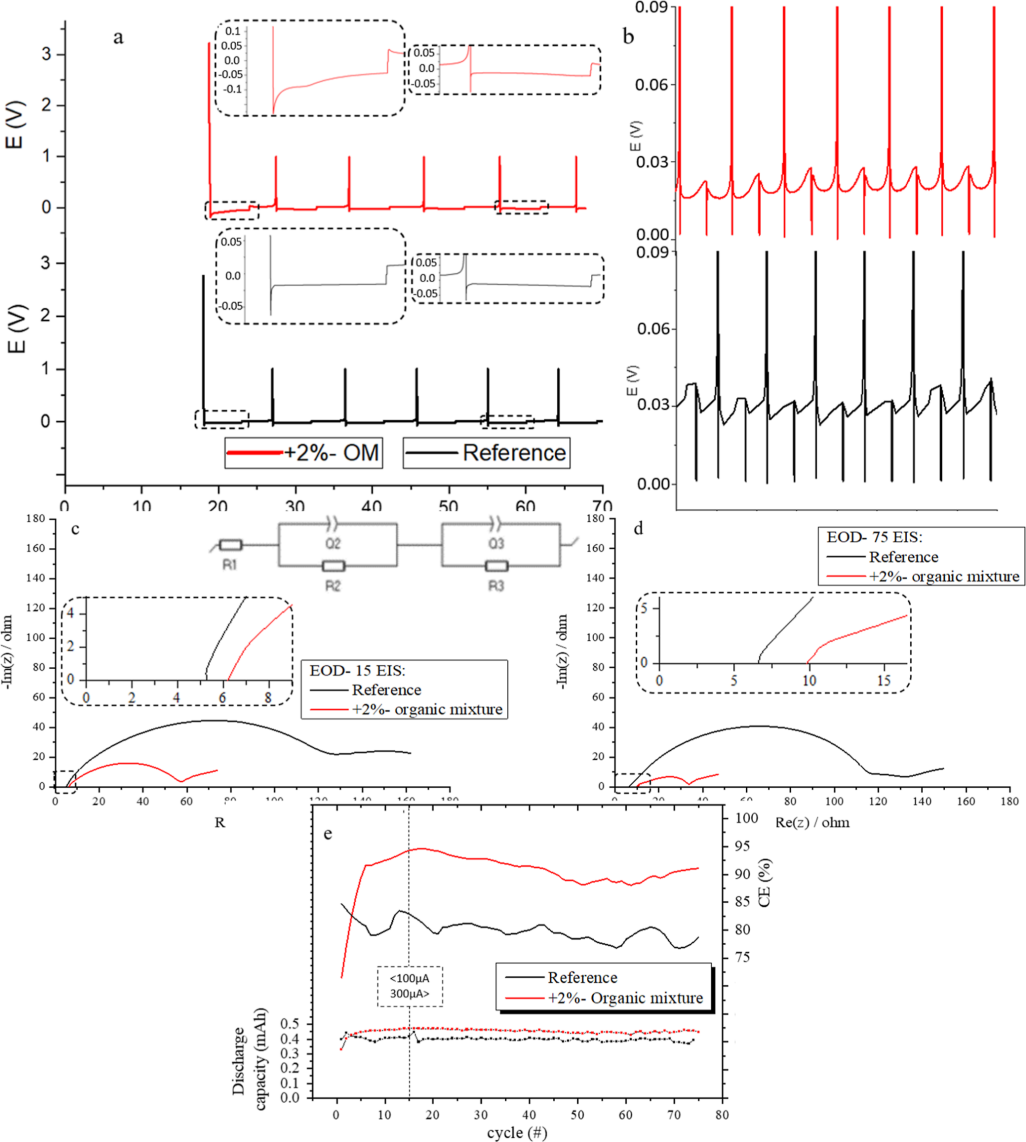
Voltage profiles for the reference cell and cell with 2% organic mixture (OM), (a) at the beginning of cycling (100 μA) and (b) at mature cycles (300 μA) (1.0 M LiPF6 dissolved in the EC:DEC 1:1 electrolyte, Li||Cu). EIS Nyquist spectra (c) at cycle 15 and (d) at cycle 75 (after full discharge), for the reference cell and cell with 2% organic mixture (OM) additives, in 1.0 M LiPF6 dissolved in the EC:DEC 1:1 electrolyte. The equivalent circuit that was used in the spectra fitting is shown in (c). (e) Summary of CR and CE parameters for the cells with organic mixture (OM) additives.

Li/Cu cells were discharged for 0.5 mA h (lithium deposition) and were charged (lithium dissolution) to 1.0 V. The applied current was 100 μA for the first 15 cycles and was raised to 300 μA for the rest of the cycles, 75 cycles overall. The effect of the OM addition on the cycling performance and the SEI properties are reported. The voltage profiles are presented in Figure 1a,b. The first part of cycling (low current density, 100 μA) and the second part (high current density, 300 μA) are presented in Figure 1a,b, respectively. The main differences between the reference and the OM cells are observed in the first cycle and during mature cycles. In the first cycle, higher overpotential (OP) is observed for the OM cell, where the negative peak at the beginning of deposition is −0.18 V and the plateau under zero starts at −0.05 and ends at −0.05 V, while for the reference cell, these values were −0.06 V and −0.015 to 0.012 V, respectively. After the first cycle, similar voltage profile wave forms are observed for both the reference and the OM cells (Figure 1a). In matured cycles and after the current density was raised, the voltage profile wave forms for the OM cells are more uniform and with lower OP. For the reference cells, the wave forms are nonuniform and with rapid cutoff voltage changes around 0.03 V, while for the OM cell, the changes are around 0.02 V and the wave forms are more uniform and with minor changes in the cutoff voltage (Figure 1b). Electrochemical impedance spectroscopy (EIS) was conducted at the end of the 15th and 75th discharges. The OM cell presented much lower resistance values compared to the reference cell at cycle 15 and at cycle 75 (Figure 1c,d and Table S1). EIS conducted at the end of the 15th discharge (EOD-15) revealed a significant reduction to charge transfer (*R*_CT_) from 129.9 Ω for the reference cell to 50.9 Ω for the OM-based cell. The resistance of the SEI (*R*_SEI_) for the OM cell is 2.0 Ω less than that of the reference cell and *R*_b_ (bulk or electrolyte resistance) is 1.1 Ω greater for the OM cell (Table S1a). As for the EIS conducted at the end of the 75th discharge (EOD-75), significant changes are observed, as well. The resistance of the electrolyte (*R*_b_) is 6.6 Ω for the reference cell and 9.9 Ω for the OM cell. OM molecules are large molecules that could interfere with Li^+^ migration through the electrolyte. *R*_SEI_ is similar for both cells with 4.9 Ω for the reference cell and 4.1 Ω for the OM cell. The value with the most significant difference between the cells is *R*_CT_ (charge-transfer resistance), which exists as the last semicircle component in the Nyquist spectra. For the reference cell, *R*_CT_ is 108.1 Ω, and for the OM cell, it is 20.0 Ω, which is a 500% lower value (Table S1b).

The efficiency of the battery in terms of current was improved by the addition of 2%-OM. For the reference cell, only 80% of the charge received by the cell was available to be discharged. For the OM cell, the CE reached 91%, an 11% improvement (Figure 1e and Table S2a). Furthermore, the irreversible capacity (*Q*_ir_), which is the CE % in the first cycle, is very different between the two cells. For the OM cell, the CE % in the first cycle is 66.0%, and for the reference cell, it is 79.3%, which corresponds to *Q*_ir_ of 34.0% and 20.7%, respectively, one and a half times higher for the OM cell (more capacity loss at the first cycle for the OM cell). This might indicate a thicker SEI generated in the first cycle, subsequent to the reduction of the additives. The fact that after the first cycle, the CE % is higher for the OM cell reflects on a more efficient Li transport through the SEI and lower “dead-Li” formation for the OM cell.

**Figure 2:**
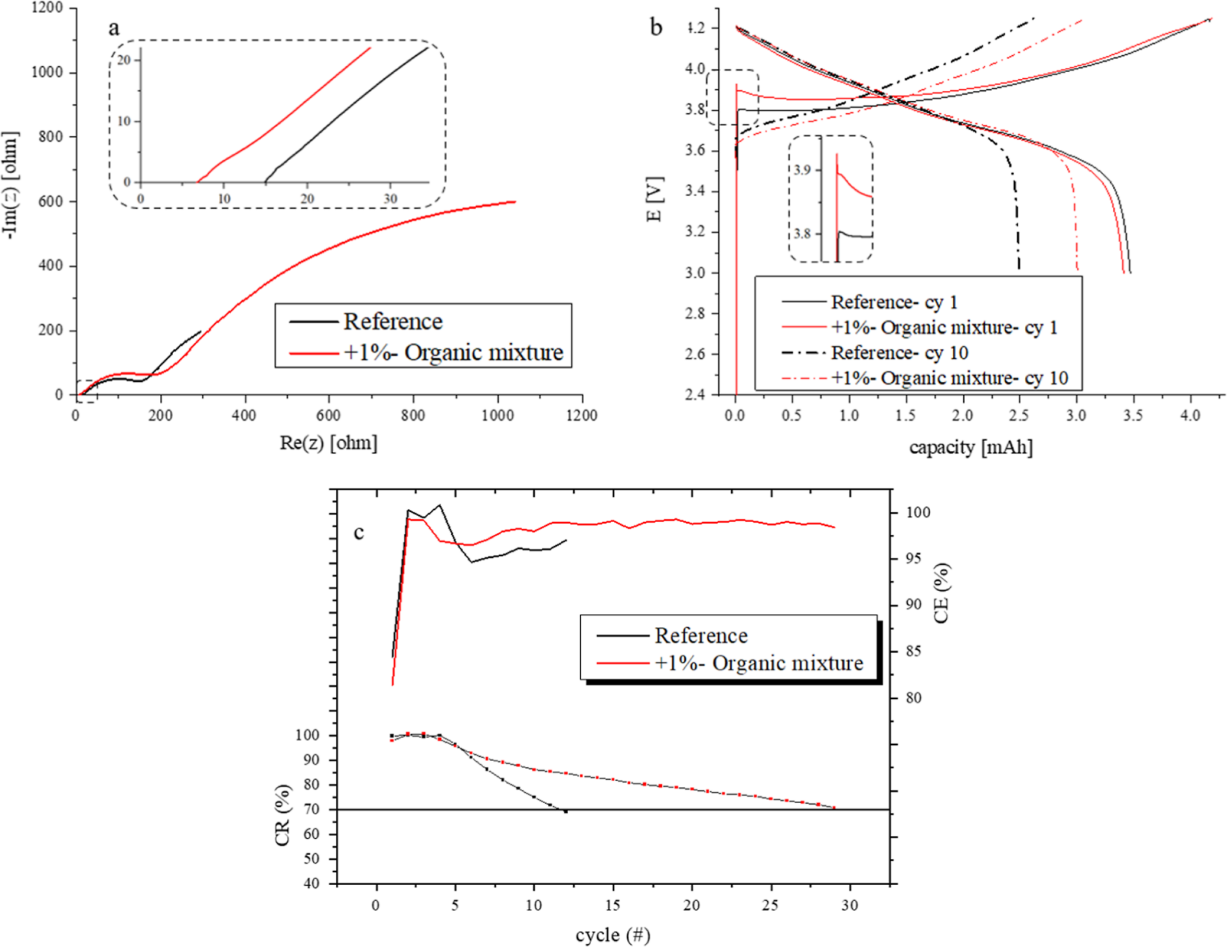
(a) EIS Nyquist spectra, at cycle 70% CR (after full discharge), for the reference and +1% organic mixture (OM). (b) Potential vs capacity graph for the reference and for +1%-OM cells at the 1st cycle and at the 10th cycle. All in AFLMB configuration. (c) Summary of CR and CE parameters for the cells with organic mixture additives in 0.95 M LiPF_6_ + 0.05 M LiBOB dissolved in the EMC:DMC:FEC:PC 3:3:3:1 electrolyte (NCA||Cu).

The AFLMB reference cell lost 30% of its initial capacity after 12 cycles, with an average CE of 97.1%. For the OM cell, a cycle of 70% CR was obtained after 30 cycles (nearly three times improvement compared to the reference cell) with an average CE of 98.5% (1.4% improvement compared to the reference cell) (Figure 2c and Table S2b).

EIS measurements were carried out at the end of discharge at cycle 70% CR. Figure 2a presents the Nyquist plots for the cells with and without OM addition. Table S3 presents the values for the bulk resistance (*R*_b_), SEI resistance (*R*_SEI_), and charge-transfer resistance (*R*_CT_) obtained from equivalent-circuit fittings and presents the CE % and cycle 70% CR. *R*_b_ is the highest for the reference cell with 15.0 Ω while *R*_b_ is 6.6 Ω for the OM additives cell. The effect on *R*_SEI_ is even more pronounced as the reference cell obtained 49.4 Ω which is more than four times larger than for cells containing OM additives (Figure 2a and Table S3).

The voltage versus capacity graph emphasizes, again, the superiority of OM-containing cells, as greater capacity is obtained for a given voltage, and improved cyclability is achieved, as could be seen in Figure 2b. At the first cycle, greater activation OP is seen for the OM-containing cell, but this trend is reversed as could be seen in the tenth cycle voltage vs capacity graph (Figure 2b).

**Figure 3:**
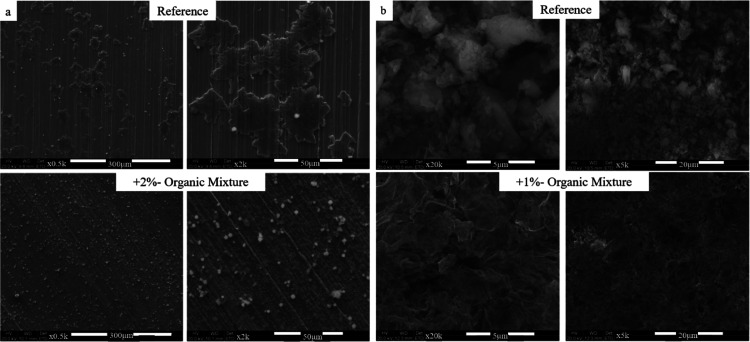
ESEM images of the anode (copper side), extracted from the (a) Cu||Li cell, after one charge of 0.1 mA h/cm^2^, for the reference and for +2%-organic mixture (OM) cells. Electrolyte composition was 1 M LiPF_6_ in 1:1 EC:DEC, and (b) Cu||NCA cell at cy 70% CR, for the reference and for +1%-organic mixture (OM) cells. Electrolyte composition was 0.95 M LiPF_6_ + 0.05 M LiBOB dissolved in EMC:DMC:FEC:PC 3:3:3:1.

Figure 3a presents SEM images of the anodes (copper with the SEI on top) of the reference cell and the cell containing 2%-OM addition, in 1 M LiPF_6_ dissolved in 1:1 EC:DEC, extracted from Cu vs Li cells, after one partial deposition of 0.1 mA h cm^–2^. The morphology of the initial Li deposits (along with the initial SEI) for the OM-containing cells looks rougher (higher surface area) and with more nucleation sites on the Cu, compared with the reference cell. In accordance, for the reference cell, less Cu is covered with Li and the SEI, and the Cu electrode seems cleaner and exposed. The SEI/Li existing on the Cu extracted from the OM-containing cell is more scattered and uniform. The SEM image observation implies the OM-additives cycling improvement mechanism, as Cu is a good electronic conductor and electrons passing through the Cu into the electrolyte would likely lead to electrolyte reduction or to an uneven Li deposition that could result in a quicker cell drying and failure. The better coverage keeps the electrolyte from being reduced.

Figure 3b presents SEM images of the anodes (copper) of the reference cell and the cell containing 1%-OM addition, extracted from Cu vs NCA cells, after 30% capacity loss at a discharged state. Electrolyte composition was 0.95 M LiPF_6_ + 0.05 M LiBOB dissolved in EMC:DMC:FEC:PC 3:3:3:1. The morphology of the SEI extracted from OM-containing cells shows a smoother and more uniform surface as well as a less dendritic morphology, which appears dendritic, compared to the reference cell.

**Figure 4:**
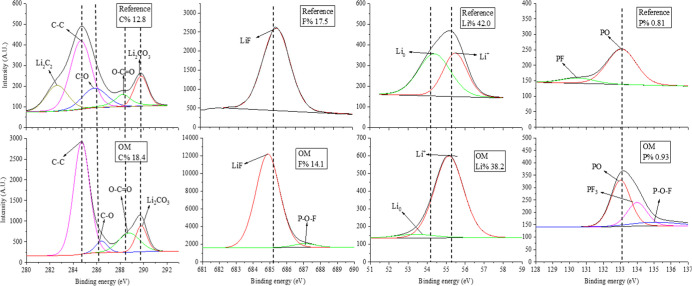
XPS spectra of the SEI (on Cu) for the reference cell and +1% organic mixture (OM), after cycle 70% CR at the discharge state, after 10 min of sputtering, for C 1s, Li 1s, F 1s, and P 2p spectra, extracted from the Cu||NCA cell. Electrolyte composition was 0.95 M LiPF_6_ + 0.05 M LiBOB dissolved in EMC:DMC:FEC:PC 3:3:3:1.

XPS measurements were conducted on the cycled copper current collectors for the reference cell and for the 1%-OM cell, without exposure to air. The copper was taken after complete dissolution of lithium (end of discharge) and after cycle 70% CR. Figure 4 presents the XPS spectra for F, C, Li, and P elements after 10 min of sputtering (inner SEI, near the SEI/Cu interphase).

Perhaps the most important XPS spectra, Li 1s, reveal the absence of “dead Li” in the SEI of OM-containing cells. For the reference cell, two peaks with the same intensity are observed, one at 54.3 eV which is attributed to metallic Li (“dead Li”) and another observed for both SEIs, at 56.0 eV, attributed to Li^+^ moieties, mainly LiF and Li_2_O.^26^ The last observation shows that less “dead Li” forms in the SEI of OM-containing cells, emphasizing the improved performance of cells containing OM additives. Moreover, the amount of Li in the SEI of the OM cell is smaller compared to the reference cell (38.2% and 42.0%, respectively), indicating less “dead-Li” is being accumulated in the SEI during operation of the cell.

F 1s spectra (Figure 4) are similar between the two cells with a peak for LiF at 684.9 eV.^26^ For the  1%-OM cell, another peak is observed at 687.0 eV, which is attributed to the P–O–F group, also observed at the P 1s spectra at 135.0 eV.^27^ The amount of F in the SEI of the OM-containing cell is 14.1%, which is lowered compared to the reference cell with 17.5%. This might be caused because the OM cell covers the anode more efficiently, decreasing the amount of electrolyte F-containing reduction products in the SEI.

P 2p spectra (Figure 4) imply chemical decomposition of the anion, PF_6_^–^. The reference cell presents a spectrum which implies that the anion undergoes greater decomposition and its reduction products are found in the SEI, as its peak at 133.0 eV which is attributed to P–O is very large and the peak at 130.5 eV^27^ attributed to the P–F bond is very small.^28^ The main P species in the SEI for the reference cell is P–O. On the other hand, for the OM-containing cell, three peaks are observed: the same P–O peak but not the peak for P–F. Instead of the last, two peaks attributed to PF_3_ and P–O–F are observed at 134.0 and 135.1 eV.^29^ This implies that the addition of OM additives slows the anion decomposition as the intermediate components are observed in the SEI of the OM cell. The amount of P in the SEI of the OM cell is 0.93%, which is higher compared to the reference cell with 0.81%.

As for the C 1s spectra (Figure 4), the two cells present the same four peaks attributed to C–C, C–O, O=C–O, and Li_2_CO_3_ at 284.7, 286.5, 288.8, and 292.0 eV, respectively.^26^ For the reference cell, an additional peak is observed at 282.9 eV, attributed to Li_2_C_2_.^26^ Li_2_C_2_ formation results from further reduction of Li_2_CO_3_,^29^ and thus, the observation implies that OM addition stops or slows the reduction of Li_2_CO_3_. The amount of C in the SEI of OM cells (18.4%) is greater compared to the amount of C in the SEI of the reference cell (12.8%). An organic-rich SEI with enhanced properties is created on the surface of the Cu foil due to the presence of OM in the electrolyte.

Figure S1 presents the O 1s spectra and strengthens the conclusion that the generated SEI is less inorganic and more organic for the OM-containing cells. Two peaks are observed for both cells at 531.2 and 528.4 eV, which correspond to C–O and LiO_2_.^26^ For the reference cell, the intensities ratio between C–O and LiO_2_ is two, and for the OM cell, it is roughly ten, emphasizing the organic nature of the SEI generated with the presence of organic additives.

**Figure 5:**
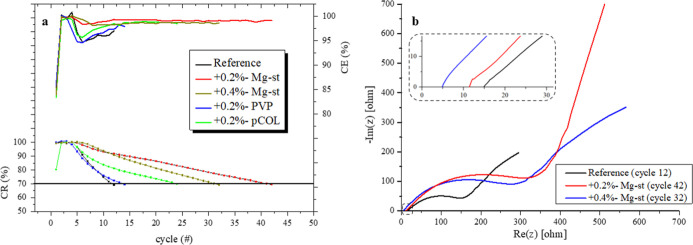
(a) CR and CE parameters and (b) EIS Nyquist spectra, at cycle 70% CR (after full discharge), for the reference cell and for cells with sole organic additive, in 0.95 M LiPF_6_ + 0.05 M LiBOB dissolved in the EMC:DMC:FEC:PC 3:3:3:1 electrolyte. All in AFLMB configuration.

It was further observed that each of the three components of the OM (pCOL, PVP and Mg-st) has had a positive effect on the cyclability of the AFLMB. The most significant improvement was found for a cell with the addition of 0.2%-Mg-st, with 70% CR after 42 cycles, 3.5 times higher compared to the reference cell with a value of 12 cycles. The average CE % of the 0.2%-Mg-st cell was 99.1%, 2% more compared to the reference cell with a value of 97.1%. Addition of 0.4%-Mg-st resulted in a moderated improvement, where the cell had lost 30% of its initial capacity after 32 cycles with an average CE % of 98.7%. 0.2%-pCOL resulted in a 2-fold improvement at a cycle of 70% CR after 24 cycles with the CE % equal to 98.3%. The addition of PVP had a minor effect on the cyclability of the cell (Figure 5a and Table S4).

Figure 5b presents the EIS Nyquist spectra for the reference and Mg-st cells at a discharged state at the cycle of 70% CR. The summary table of the extracted resistance values is presented in Table S5. Addition of 0.2%-Mg-st resulted in a 50% decrease in *R*_b_, from 15.0 to 10.6 Ω compared to the reference cell, and, interestingly, addition of twice the amount (0.4%) resulted in another 50% decrease, which is 4.9 Ω. Regarding *R*_SEI_, addition of 0.2% Mg-st resulted in a 50% decrease, from 49.4 to 25 Ω. Adding twice the amount (0.4%-Mg-st) resulted in a very low value of 6.1 Ω. At the higher concentration, more hydrocarbons from the stearate molecule are present in the electrolyte and could be reduced on the anode and become a part of the SEI, containing long organic legs toward the electrolyte. *R*_CT_ was very high for the Mg-st cells, almost twice the value for 0.2% addition (414.7 Ω), and for 0.4% addition, the *R*_CT_ value was 344.4 Ω compared to 214.4 Ω for the reference cell.

## Conclusions

This work explores three organic materials (Mg-st, PVP, and pCOL) as low-concentration electrolyte additives for LMBs. The systems investigated were Li-metal||Cu and NCA||Cu. It was found that the addition of OM affects many properties of the cell. For the Li-metal configuration, EIS tests revealed that charge-transfer resistance is 540% lowered for the OM-containing cell compared to the reference cell after 75 cycles of Li deposition/dissolution on Cu. The resistance of the SEI was also reduced in a more moderate way, while the resistance of the bulk increased by 3.3 Ω. The addition of OM in this configuration (Li-metal||Cu) increased the current efficiency (CE) by more than 10% from 79.9% for the reference cell to 91.2% for the OM-containing cell. It was found that the coverage of the anode (Cu foil) is much higher for the OM-containing cell after the initial 0.1 mA h cm^–2^ deposition, resulting in less Li consumption.

For the AFLMB configuration (NCA||Cu), XPS spectra revealed that a very small amount of “dead-Li” is present in the SEI of the OM-containing cell compared to the reference cell. The resistance of the SEI in this configuration is 3.8 times lower for the OM-containing cell, and the resistance of the bulk is reduced by more than two times. SEM imaging showed that the morphology of the anodic SEI is much smoother and uniform for cells with organic additives.

It was also shown that using each of the components of the OM additives further increased the cyclability of the AFLMB configuration and reached among the best life performances in the literature for similar systems with high Li capacities.^14,30,31^

As shown in this work, addition of OM as additives to the LMB could increase their safety and life span, possibly through reduction of organic molecules to form an organic-rich SEI with enhanced properties, which increases the efficiency of Li diffusion through the formed layer. This work opens the door for future research on similar organic materials in alkali metal batteries for energy storage systems.

## Supporting Information Available

Summary tables for cycle life performance, summary tables for EIS, and O 1s XPS spectra of the SEI (PDF)
