# Supplementary material for: Improvement of Lithium-Metal Batteries by Addition of a Low Concentration of Organic Molecules
Source: ACS Appl Mater Interfaces. 2025 May 1;17(19):28822–9. doi: 10.1021/acsami.5c01585 (PMC12086764; doi:10.1021/acsami.5c01585)
Supplement: Supplementary file 1 — am5c01585_si_001.pdf [file am5c01585_si_001.pdf]

Supporting Information

Improvement of Lithium-Metal Batteries by Addition of a Low Concentration of Organic Molecules

*Roy Marrache*** *and Emanuel Peled**

School of Chemistry, Faculty of Exact Sciences, Tel Aviv University, Tel Aviv, 69978, Israel

**School of Chemistry, Faculty of Exact Sciences, Tel Aviv University, Tel Aviv, 69978, Israel*

*peled@tauex.tau.ac.il , **roymarrache@mail.tau.ac.il

- Summary tables for cycle life performance.
- Summary tables for EIS.
- O1s XPS spectra.

a

R table (ohm)	End of the 15th discharge			Cycling performance
**Additive type**	R_b	R_{SEI}	R_{CT}	**CE%**
**reference**	5.3	8.5	129.9	79.9
**+2%- OM**	6.4	6.5	50.9	91.2

b

R table (ohm)	End of the 75th discharge			Cycling performance
**Additive type**	R_b	R_{SEI}	R_{CT}	**CE%**
**Reference**	6.6	4.9	108.1	79.9
**+2%- OM**	9.9	4.1	20.0	91.2

Table S1- EIS resistance values obtained from Nyquist spectra, at cycle 15 (a) and at cycle 75 (b), after full discharge, for the reference cell and cell with 2% organic mixture (OM), (1.0M $LiPF_6$ dissolved in EC:DEC 1:1 electrolyte, Li||Cu).

a

Additive	CE- 1st cycle (Qir)	CE- 2nd- 75th cycle
**Reference**	79.3	79.9
**+2%- OM**	66.0	91.2

b

Additive	CE- 1st cycle (Qir)	CE- 2nd- cycle 70% CR	Cycle of 70%CR	Loss per cycle (%)
**Reference**	84.4	97.1	12	2.8
**+1%- OM**	81.4	98.5	30	1.0

Table S2- Summary of CR and CE parameters for the cells with and without OM addition. (a) 2%- OM , in 1.0M $LiPF_6$ dissolved in EC:DEC 1:1 electrolyte (Li||Cu cell) and, (b) 1%- OM in 0.95M $LiPF_6$ + 0.05M LiBOB dissolved in EMC:DMC:FEC:PC 3:3:3:1 electrolyte (NCA||Cu cell).

R table (ohm)	Cycle 70%CR			Cycling performance
**Additive type**	R_b	R_{SEI}	R_{CT}	**CE%**
**Reference**	15.0	49.4	214.4	97.1
**+1%- OM**	6.6	12.9	215.0	98.5

Table S3- summary table of resistance values retrieved from EIS, at cycle 70%CR (after full discharge), for the reference and +1%- organic mixture (OM) cells (NCA||Cu).

Additive	CE- 1st cycle (Qir)	CE- 2nd- cycle 70% CR	Cycle of 70%CR	Loss per cycle (%)
**Reference**	84.4	97.1	12	2.8
**+0.2%- Mg-st**	83.7	99.1	42	0.7
**+0.4%- Mg-st**	83.9	98.7	32	0.9
**+0.2%- pCOL**	83.4	98.3	24	1.2
**+0.2%- PVP**	85.7	97.0	14	2.1

Table S4- Summary of CR and CE parameters for the cells with and without organic additives, in 0.95M $LiPF_6$ + 0.05M LiBOB dissolved in EMC:DMC:FEC:PC 3:3:3:1 electrolyte (NCA‖Cu configuration).

R table (ohm)	Cycle 70%CR			Cycling performance
**Additive type**	R_b	R_{SEI}	R_{CT}	**CE%**
**Reference**	15.0	49.4	214.4	97.1
**+0.2%- Mg-st**	10.6	25.0	414.7	99.1
**+0.4%- Mg-st**	4.9	6.1	344.4	98.7

Table S5- summary table of resistance values retrieved from EIS, at cycle 70%CR (after full discharge), for the reference, +0.2%- Mg-st and +0.4- Mg-st in 0.95M $LiPF_6$ + 0.05M LiBOB dissolved in EMC:DMC:FEC:PC 3:3:3:1 electrolyte (NCA||Cu configuration).

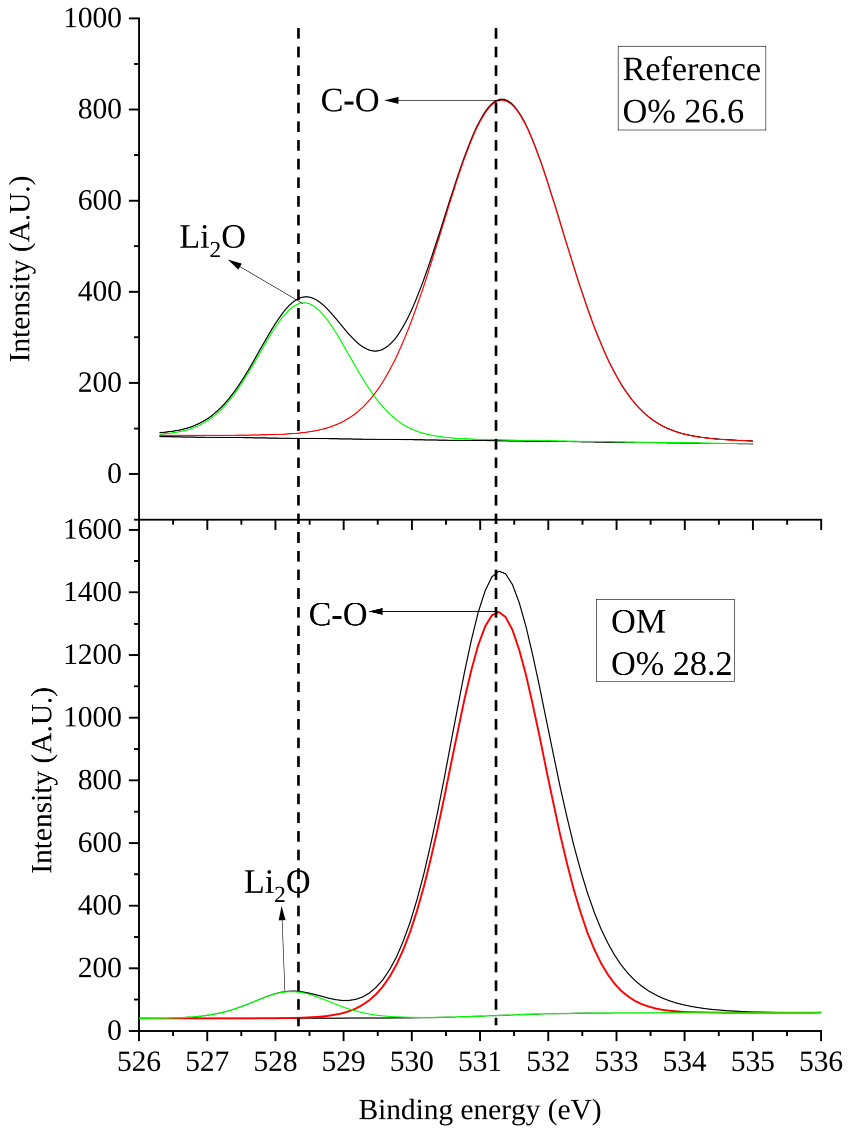

Figure S1- XPS O1s spectra of the SEI (on Cu anode) for reference cell and +1%- organic mixture (OM), after cycle 70% CR at discharge state, after 10 min of sputtering, extracted from Cu || NCA cell. electrolyte composition was 0.95M $LiPF_6$ + 0.05M LiBOB dissolved in EMC:DMC:FEC:PC 3:3:3:1.
